# The impact of exercise training on calf pump function, muscle strength, ankle range of motion, and health-related quality of life in patients with chronic venous insufficiency at different stages of severity: a systematic review

**DOI:** 10.1590/1677-5449.200125

**Published:** 2021-04-28

**Authors:** Keity Lamary Souza Silva, Eduardo Augusto Barbosa Figueiredo, Cíntia Pimenta Lopes, Marcus Vinicius Accetta Vianna, Vanessa Pereira Lima, Pedro Henrique Scheidt Figueiredo, Henrique Silveira Costa

**Affiliations:** 1 Universidade Federal dos Vales do Jequitinhonha e Mucuri – UFVJM, Faculdade de Ciências Biológicas e da Saúde, Departamento de Fisioterapia, Diamantina, MG, Brasil.; 2 Universidade Federal dos Vales do Jequitinhonha e Mucuri – UFVJM, Faculdade de Medicina, Diamantina, MG, Brasil.; 3 Universidade Federal dos Vales do Jequitinhonha e Mucuri – UFVJM, Programa de Pós-graduação em Reabilitação e Desempenho Funcional, Diamantina, MG, Brasil.

**Keywords:** venous insufficiency, exercise, muscle strength, articular range of motion, quality of life, insuficiência venosa, exercício físico, força muscular, amplitude de movimento articular, qualidade de vida

## Abstract

Exercise training (ET) is an important tool in the management of patients with chronic venous insufficiency (CVI). The objective of this article was to discuss the effects of ET on the calf pump, functional parameters, and quality of life of patients with mild and advanced CVI. A systematic review was conducted and eleven studies were included. In patients with mild CVI, ET was effective for improving venous reflux, muscle strength, ankle range of motion, and quality of life. In advanced CVI patients, ET increased ejection fraction, reduced residual volume fraction, and improved muscle strength and ankle range of motion, but did not change venous reflux indices or quality of life. It is concluded that ET is effective for improving calf pump function, muscle strength, and ankle range of motion in CVI. In patients with mild CVI, additional benefits were observed in quality of life.

## Resumo

O treinamento físico é uma importante ferramenta no tratamento de pacientes com insuficiência venosa crônica. O objetivo foi discutir os efeitos do tratamento físico na bomba da panturrilha, os parâmetros funcionais e a qualidade de vida de pacientes com insuficiência venosa crônica leve e avançada. Uma revisão sistemática foi realizada, e 11 estudos foram incluídos. Na insuficiência venosa crônica leve, o treinamento físico foi eficaz na melhora do refluxo venoso, da força muscular, da amplitude de movimento do tornozelo e da qualidade de vida. Na insuficiência venosa crônica avançada, o treinamento físico aumentou a fração de ejeção, reduziu a fração de volume residual e melhorou a força muscular e amplitude de movimento do tornozelo, sem alterações nos índices de refluxo venoso e na qualidade de vida. Conclui-se que o treinamento físico é eficaz na melhoria da bomba da panturrilha, da força muscular e da amplitude de movimento do tornozelo na insuficiência venosa crônica. Em pacientes com insuficiência venosa crônica leve, foram encontrados benefícios adicionais na qualidade de vida.

## INTRODUCTION

Chronic venous insufficiency (CVI) is characterized by abnormal lower limb venous system function. It has a high prevalence and morbidity in Brazil^1^^,^ 2 and worldwide,^3^ with considerable economic impact due to disability.^4^ Previous studies have estimated that 5 to 30% of the adult population have signs or symptoms of the disease and CVI can therefore be considered a serious public health problem.^5^^,^ 6

Chronic venous insufficiency has a broad spectrum of clinical manifestations, ranging from telangiectasias and reticular veins to active and recurrent venous ulcers.^7^ The pathophysiologic mechanism of the disease is multifactorial^8^ and may be due to mechanical obstruction of the venous flow (superficial and deep), valve incompetence, or dysfunction of the calf muscle pump.^8^^-^ 10 There is an imbalance of blood flow in the lower limbs that compromises venous return,^2^ resulting in increased venous pressure.^10^ Consequently, many studies have shown the harmful consequences of CVI,^1^ such as impaired work activities, poor performance in daily activities, and decline in emotional health and self-esteem.^1^^,^ 6 ^,^ 11 It has also been linked to worse health-related quality of life^12^^,^ 13 due to pain, leg tiredness, edema and, in advanced cases, ulcerations.^2^^,^ 9 In this scenario, exercise training (ET) emerges as an important tool in the conservative management of patients.^14^ Several studies have reported that ET prevents the progression and complications of venous disease, reduces symptoms and improves health-related quality of life.^5^^,^ 15

However, data about the benefits of ET is scattered and needs to be systematically discussed to increase knowledge about exercise prescription in these patients. Two well-designed systematic reviews were addressed to verify the effect of ET on patients with CVI. One^16^ demonstrated that ET is effective in improving some parameters of calf pump function, such as the ejection fraction, but not the ankle range of motion or healing of venous leg ulcers. However, the review included studies with patients at different stages of the disease, i.e., with or without venous leg ulcers. Another review^4^ included studies that only encompassed patients without venous leg ulcers. Considering the heterogeneous clinical, functional, and health-related quality of life expression of mild patients compared to those with skin changes, it is necessary to verify the effect of ET at different stages of the disease. Therefore, the present study aimed to systematically discuss the effects of ET on variables related to calf pump function, functional parameters, and health-related quality of life in patients with mild and advanced CVI.

## METHODS

### Study design

This systematic review aimed to verify the impact of ET on calf pump function, functional parameters, and health-related quality of life of patients with CVI. The study was registered on the PROSPERO database (CRD42020159204) and edited following the guidelines of the Preferred Reporting Items for Systematic Reviews and Meta- Analyses (PRISMA) statement.^17^

### Search strategy and study selection

Potential studies were identified using a systematic search strategy. The databases MEDLINE, Web of Science, Cumulative Index to Nursing and Allied Health Literature (CINAHL), Latin American & Caribbean Health Sciences Literature (LILACS), Scopus, and Physiotherapy Evidence Database (PEDro) were searched for relevant studies, with no date restrictions, from inception up to May 2020. Searches were conducted independently by 2 authors (CPL and HSC) from May to June of 2020. Disagreements were resolved by a third reviewer (KLSS). Search terms included words related to chronic venous insufficiency, venous leg ulcer, and exercise training. The following strategy was used for the PubMed search: (“venous insufficiency” OR” venous disease” OR “chronic venous disease”) AND (“exercise” OR “physiotherapy” OR “physical therapy”) and was modified to suit each database.

### Eligibility criteria

The eligibility criteria were studies that a) evaluated patients diagnosed with CVI; b) performed ET in patients with CVI; and c) used calf pump parameters, functional variables, and health-related quality of life as outcomes. Exclusion criteria were review studies, articles in duplicate, animal studies, and those that did not fit the objective of this review. Papers dealing with post-thrombotic syndrome, that combined non-conservative treatment techniques, and/or used other modalities of physical therapy, such as balneotherapy and manual therapy, were also excluded.

### Quality assessment

Quality was assessed using the PEDro Scale, described in the Physiotherapy Evidence Database (www.pedro.org.au). This scale consists of 11 items and was developed to classify the methodological quality (internal validity and statistical information) of randomized clinical trials. Studies not validated by the PEDro scale were evaluated by the authors.

### Outcomes and data analysis

The following data were extracted from the included articles: author, publication year, characteristics of the sample (both experimental and control group, if applicable), treatment, and changes in the parameters evaluated before and after exercise training. Patients with CVI were stratified according to CEAP class,^7^ as follows: telangiectasias or reticular veins (C1); varicose veins (C2); recurrent varicose veins (C2r); edema (C3); changes to skin and subcutaneous tissue secondary to CVI (C4); pigmentation or eczema (C4a); lipodermatosclerosis or atrophie blanche (C4b); corona phlebectatica (C4c); healed venous ulcer (C5); active venous ulcer (C6); or recurrent active venous ulcer (C6r). Due to the differences in clinical expression with functional impact, studies were stratified as investigating mild CVI (CEAP class less than or equal to 3) or advanced CVI (with skin abnormalities and venous ulcer; CEAP class greater than or equal to 4). When a study encompassed a wide variety of CEAP classes, the one with the largest sample size was considered for stratification.

The primary outcome measure was intragroup or intergroup difference in calf pump parameters, functional variables, and health-related quality of life.

## RESULTS

### Flow of papers through the review

**Figure 1:**
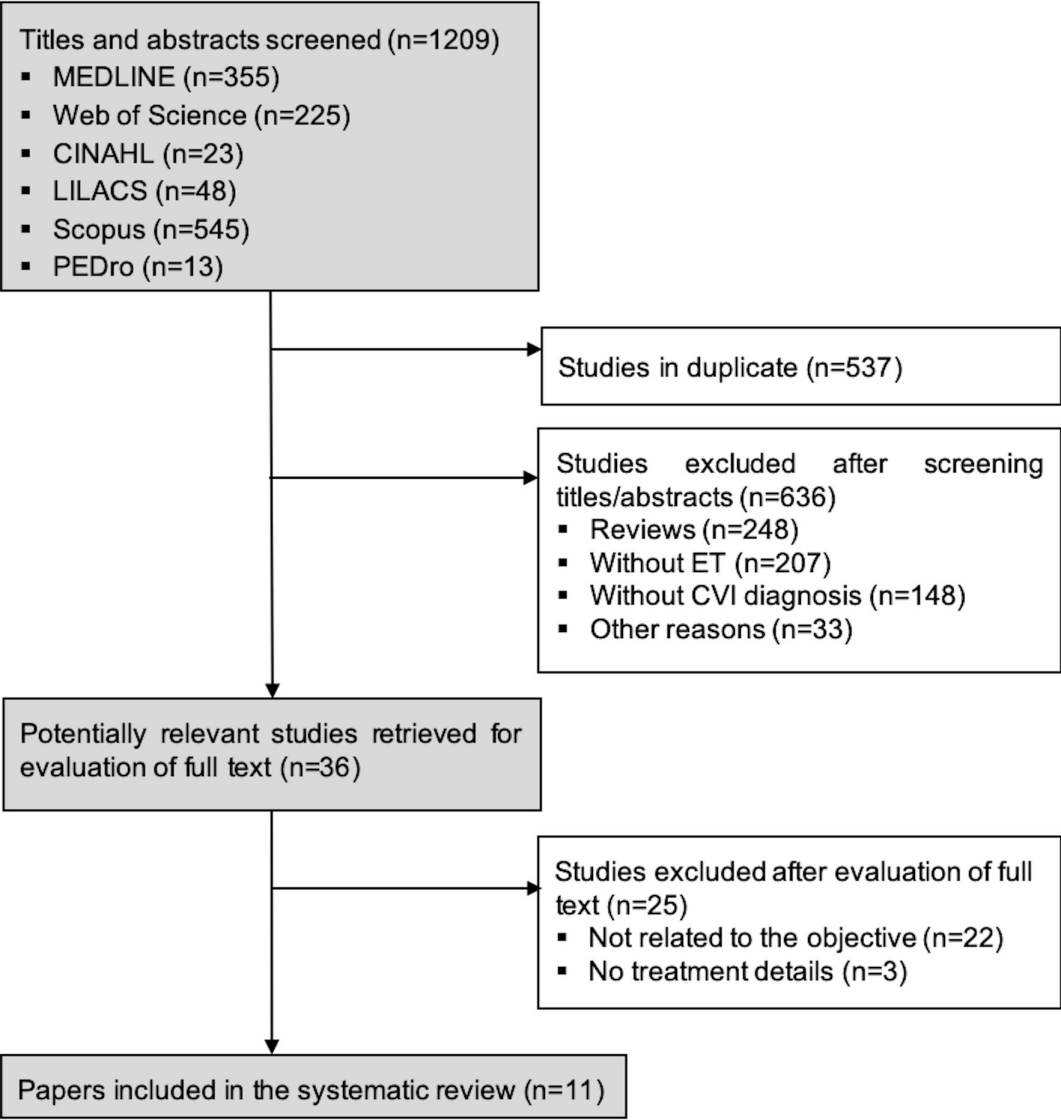
The PRISMA flow diagram of papers through the review. CVI: chronic venous insufficiency; ET: exercise training; MEDLINE: Medical Literature Analysis and Retrieval System Online; CINAHL: Cumulative Index to Nursing and Allied Health Literature; LILACS: Latin American & Caribbean Health Sciences Literature.

The electronic search strategy identified 1,186 studies, but 525 (42%) of these were duplicates. After screening titles and abstracts, 626 papers were excluded. Most of them were review studies, did not perform ET, or used a sample without CVI. After reading the full texts, 24 articles were excluded for failing to fit the objectives of the present review. A total of 11 articles were included in the present review. Figure 1 outlines the flow of papers through the review.

### Participants

Five studies investigated the effect of ET on patients with mild CVI. Three of these studies (60%) each analyzed a single group of patients, one (20%) compared the ET group with usual care, and another compared ET against ET associated with intermittent pneumatic compression. The number of sessions ranged from 10 to 48. The mean quality assessment score for the studies included was 2.8, ranging from 1 to 7.

Another seven studies aimed to verify the impact of ET on calf pump function, functional parameters, and health-related quality of life of patients with CVI with skin changes or venous leg ulcers. Three of these studies, (44%) analyzed a single group each, two (28%) had a group given usual care combined with compression as control, in one study (14%) the control group was kept on usual care, and in one study (14%) the control group was instructed to perform exercises at home, without supervision. The number of sessions ranged from 18 to 48. The mean score for methodological quality was 3.6 (ranging from 1 to 7).

### Outcomes

Nine studies demonstrated the effect of ET on calf pump function, four in mild CVI and five in patients with advanced CVI. The variables ejection fraction, residual volume fraction, residual volume, ejected volume, venous volume, venous filling time, and blood flow were used in the studies included and defined according to Jull et al.^18^

The functional parameters evaluated included those related to ankle range of motion and strength. Ankle range of motion after ET was assessed by goniometry in 4 studies, two in mild patients,^11^^,^ 12 and two in patients with advanced CVI.^19^^,^ 20 Strength was tested with isokinetic dynamometry in three studies, one in mild CVI^11^ and two in CVI with skin changes.^21^^,^ 22 The variables used were the peak torque, peak initial torque, the ratio between peak torque and body weight, and ankle total work.

Four studies evaluated the impact of exercise on health-related quality of life in patients with CVI. Two of them in mild patients^11^^,^ 12 and two in patients with advanced CVI.^13^^,^ 21 The questionnaires used in the studies were both generic (the Short-form Health Survey [SF-36] and the EuroQol 5 Dimensions [EQ-5D]), and specific (the Aberdeen Varicose Veins Questionnaire [AVVQ] and the Chronic Venous Insufficiency Questionnaire [CIVIQ]).

### Outcomes in patients with mild CVI

The impact of ET on calf pump function in patients with mild CVI was verified in four studies.^11^^,^ 14 ^,^ 23 ^,^ 24 All of them demonstrated a significant improvement in venous reflux in terms of reduced venous refilling time. Improvements were also demonstrated in venous volume^14^ and blood flow.^23^

With regard to evaluation of functional parameters, two studies verified the effect of ET on the ankle range of motion^11^^,^ 12 and one^11^ verified the impact of ET on muscle strength. Ankle range of motion significantly increased after ET in both studies. Likewise, ET was also effective for increasing peak torque, peak initial torque, and total work (p<0.001 for all outcomes).

Improvements in health-related quality of life after ET were demonstrated in both studies that analyzed this outcome,^11^^,^ 12 according to the SF-36 (p <0.05 in all domains), the AVVQ (p <0.05 in all domains), and the EQ-5D (p=0.001). The main results of the effects of ET on the variables analyzed in patients with mild CVI are shown in Table 1.

**Table 1:** The effect of exercise training in management of patients with mild chronic venous insufficiency (n=5).

**Study**	**Population**	**Intervention**	**Comparison**	**Outcomes**	**PEDro Score**
Hartmann et al.^14^	12 patients (CEAP C2) with varicose veins in both extremities.	24-weeks of physiotherapy, twice a week, 60 minutes each session. The sessions consisted of bathing and 25 minutes of exercise for muscle and joint activation with compression stockings. Additionally, home-based exercises once a day for 15 minutes, wearing compression stockings	Usual care (n=12)	In the experimental group, ET reduced venous capacity (p<0.005) and venous refilling time in the lower limbs (p<0.001).	4/10
				There were no changes in the control group.	
Zajkowsk et al.^24^	11 patients (mean age 60 years), comprising CEAP C2 (n=6) and C4 and C5 (n=5).	18 sessions (1h per session), 2-3 times a week, of calf muscle strengthening with compression stockings.	Single group.	For patients with CEAP C2: venous reflux decreased.	1/10
Leal et al.^12^	10 patients (43.1±9.4 years, CEAP C2-C3)	10 sessions of 60 minutes, 3 times a week. Each session comprised therapeutic exercises (stretching the lower limbs, strengthening the calf with 2-3 sets of 10 repetitions), walking on the treadmill for 10 minutes and manual lymph drainage.	Single group.	ET increased range of motion in dorsiflexion, plantar flexion, adduction, and abduction (p<0.05 for all) and improved quality of life in all domains of the SF-36 and the Aberdeen Varicose Veins Questionnaire (AVVQ) (p<0.05 for all domains).	1/10
Ercan et al.^11^	27 patients (48±9 years, CEAP C3-C4)	12-weeks of ET, 3 days per week. The ET comprised 3 sets of 10rep of range of motion and stretching exercises, Theraband exercises, isokinetic exercise. Additionally, stability exercise on a balance board (10 min), walking on treadmill (60% HR max, 20 min) and intermittent pneumatic compression (20 min).	Single group.	Improved range of motion in dorsiflexion (p=0.018) and plantar flexion (p=0.004), increased peak torque, peak initial torque, and total dorsiflexion and plantar flexion work (p=0.001 for all), health-related quality of life by EQ-5D (p=0.001) and venous return time (p=0.001).	1/10
Elsisi et al.^23^	25 patients with bilateral varicose veins (43.88±6.73 years, CEAP C2)	3 months of ET, including gluteal and quadriceps isometric exercises, active hip and knee flexion/extension, ankle dorsiflexion/ plantar flexion, and straight leg rising in conjunction with use of an intermittent pneumatic compression device.	ET associated with tip-toe strengthening, ergometer exercise with elastic compression stockings (n=25, 44.52±6.23 years).	Both groups had improvements in maximum blood flow, mean blood flow; and venous refilling time (p<0.05). Improvements in the experimental group were significantly higher than in the control group.	7/10

### Outcomes in patients with advanced CVI

Five studies demonstrated the effects of ET on variables related to calf pump function. All articles that evaluated the ejection fraction (n=4)^18^^,^ 20 ^-^ 22 showed a significant increase in this parameter after ET. In addition, four out of five studies^20^^-^ 22 ^,^ 24 showed that ET reduced the residual volume fraction in patients with advanced CVI. None of the studies found changes in the venous volume,^18^^,^ 21 ^,^ 22 venous filling index,^18^^,^ 20 ^-^ 22 ejected volume,^18^^,^ 21 or residual volume.^18^

Improvements in ankle range of motion were detected in two studies,^19^^,^ 20 both demonstrating significant increases in this parameter after ET. Additionally, one study^19^ demonstrated that the increase in ankle range of motion was observed in groups that performed supervised and non-supervised ET, but that the increase was greater in the group performing supervised ET. Changes in muscle strength were demonstrated in one study,^21^ in terms of a significant increase in the ratio between peak torque and body weight, whereas in another study peak torque and total work did not change after ET.^22^

Regarding health-related quality of life, one study found significant improvements in this outcome according to the CIVIQ (p<0.05),^13^ while another^21^ did not find changes when using the AVVQ and CIVIQ questionnaires and the physical function items of the SF-36. Details of the results found by the studies that analyzed patients with advanced CVI are shown in Table 2.

**Table 2:** The effect of exercise training in management of patients with advanced chronic venous insufficiency (n=7).

**Study**	**Population**	**Intervention**	**Comparison**	**Outcomes**	**PEDro Score**
Yang et al.^22^	20 patients with venous leg ulcers. Median age of 68 (range: 34 to 88) years.	6 weeks of ET comprising tip-toe exercises associated with walking and calf stretch exercises.	Single group.	Increase in ejection fraction and decrease in residual volume fraction (p<0.05). No changes in venous volume, venous filling index, peak torque, or total work	1/10
Padberg et al.^21^	Patients with skin changes (n=17, mean age of 71 years, CEAP C4- C6)	6 months of stockings combined with lower limb and trunk stretching and strengthening, with active gravity strengthening and resistive weights in two sessions per week (1 hour duration).	Usual care in addition to compression (n=13, mean age of 70 years, CEAP C4- C6).	Experimental group improved residual volume fraction (p<0.029), mean ejection fraction (p<0.026), isokinetic peak torque/body weight at both slow speed (p<0.05), and fast speed (p<0.03). No changes in venous filling index, venous volume, or ejected volume. No changes were observed health-related quality of life (according to the Aberdeen Varicose Vein Survey, the CIVIQ, and the physical function items of the SF-36).	5/10
Zajkowsk et al.^24^	11 patients (mean age 60 years), comprising CEAP C2 (n=6) and C4 and C5 (n=5).	18 sessions (1h per session), 2-3 times a week, of calf muscle strengthening with compression stockings.	Single group.	For CEAP C4-C5 patients: residual volume fraction decreased (p<0.05).	1/10
Jull et al.^18^	20 patients (54.6±19.9 years) with venous leg ulcers.	12-week home-based progressive resistance exercise program using heel rises in addition to compression.	Usual care in addition to compression (n=19, 53.3±19.9 years).	Experimental group improved ejection fraction (p<0.05). There were no significant differences between groups in venous volume, ejection volume, venous filling index, residual volume, or residual volume fraction.	7/10
Szewczyk et al.^19^	Patients with venous leg ulcers (n=16, 77.2±7.66 years, CEAP C6).	9-week supervised program of exercises (circular foot movements, lifting the body weight while standing on the toes, alternate performance of foot dorsiflexion and plantar flexion). The exercises were performed 3 times a day, in series of 15 repetitions. Moreover, all patients walked 3 km daily and additionally exercised on training bikes.	Performance of exercise without supervision (n=16, 72.3±10.13 years, CEAP C6).	Experimental group increased ankle joint mobility in dorsiflexion and plantar flexion (p<0.05 for both). The control group also increased ankle joint mobility in dorsiflexion and plantar flexion (p<0.05 for both), but improvements were greater in the experimental group (p<0.05).	5/10
O’Brien et al.^20^	4 patients (66±6 years) with active venous ulceration.	12 weeks of high-compression bandaging, leg elevation and performing leg and/or ankle exercises.	Usual care (n=3, 63.6±20 years)	Experimental group increased ejection fraction (p=0.03) and decreased residual volume fraction (p=0.03). No changes in the venous filling index (p=0.17). There were also changes in ankle range of motion (p=0.01).	5/10
Kravtsov et al.^13^	22 patients with varicose veins, CEAP C3 (n=6) and C4 (n=16).	60 days of specially designed complex of 7 exercises intended to strengthen the posterior muscle group of the lower legs and correct the step cycle.	Single group.	ET improved health-related quality of life by CIVIQ (p<0.05).	1/10

## DISCUSSION

ET may have positive effects on the neuromuscular systems of patients with CVI, due to improvements in muscle structure and reduction of blood reflux and edema.^6^ The possible effects of ET on calf pump function, muscle strength, ankle range of motion, and health-related quality of life should be systematically discussed according to the severity of CVI. Therefore, the main findings of the present systematic review were that ET: 1) improves venous reflux, ankle range of motion, muscle strength, and health-related quality of life in patients with mild CVI and; 2) improves ejection fraction, residual volume, ankle range of motion, and muscle strength in patients with advanced CVI and skin changes or leg ulcers, without changing venous reflux parameters. These findings reinforce the importance of ET in clinical management of all stages of CVI, mainly by increasing muscle strength, ankle range of motion, and calf pump function. However, at more advanced stages, ET does not seem to be effective in reducing venous reflux and there is no evidence to support improvements in health-related quality of life after ET in these patients.

Muscle dysfunction is identified as one of the etiological factors of CVI.^25^ Consequently, in mild CVI, ET emerges as a useful tool for control of the signs and symptoms of the disease as well as for prevention of disabilities due to reduction of hydrostatic pressure during movement.^6^ Exercise training based on strengthening the calf muscles seems to be able to stimulate muscle strength. Furthermore, when associated with compression techniques, ET facilitates improvement of venous reflux.^24^ The possible mechanism behind this change is activation of the deep venous system, increasing its venous capacity, and reducing venous hypertension.^14^^,^ 19 ^,^ 23 This reduction in venous hypertension, caused by the combination of exercise and venous compression, stimulates production of nitric oxide, a potent neuromodulator of venous tone, with inhibition of platelet aggregation and neutrophil adhesion, which are essential elements of hypoxia injury.^23^

The combination of increased muscle strength with reduced venous reflux can improve the ankle range of motion, since both mechanisms will lead to a reduction in edema. Increased health-related quality of life is therefore expected and was found by all studies that addressed this variable, since ET is effective in reducing the signs and symptoms of CVI.

In patients with advanced CVI, ET seems to improve muscle strength, enhancing muscle contraction and increasing peak calf muscle torque. We believe that exercise can reduce the morphological changes present in patients with CVI, such as atrophy of the gastrocnemius muscle, muscle denervation, and inflammatory cell proliferation.^26^^,^ 27 Additionally, the present study demonstrated that ET is able to improve calf muscle pump function, especially residual volume fraction,^21^^,^ 24 ejection fraction,^18^^,^ 20 ^,^ 21 and venous volume.^24^ However, the venous reflux indices do not change after ET^18^^,^ 21 because there is no significant modification to the venous wall structure capable of provoking improvement in venous reflux rates.^18^^,^ 20 In fact, it is well established that the clinical improvement in patients with CVI is related to greater venous blood ejection and improved venous function.^18^ After ET, ejection of greater blood volumes, with consequent drop in venous pressure, is to be expected because of muscle strengthening.^21^ However, in advanced CVI, there is no improvement in venous function.^18^ Therefore, venous reflux and venous hypertension remain, leading to blood stasis in the lower limbs and impairing the return of blood to the heart against gravity.^3^

Ankle range of motion is related to CVI severity^28^ and to calf pump function parameters such as ejection fraction and residual volume.^29^ Since the patients showed significant improvement in calf pump function after ET, it is to be expected that range of motion would also increase.

Regarding changes in health-related quality of life, the results of the two studies that measured it in these patients were inconsistent. While one study found a significant improvement in health-related quality of life assessed by CIVIQ after ET, a clinical trial showed no improvement in the health-related quality of life evaluated by Aberdeen, CIVIQ, and SF-36 questionnaires. However, it is woth noting that the SF-36 is a generic questionnaire and does not cover the peculiarities of this disease, while the two specific questionnaires target patients with mild CVI.^30^ Therefore, the effect of ET on health-related quality of life in severe CVI remains unknown and we believe that further studies with questionnaires aimed at this specific population are desirable.

The present study has some limitations. Many studies had low quality scores, mainly due to absence of control groups. Additionally, many studies also combined compression techniques with ET, making it difficult to verify the effect of each treatment technique in isolation. One strength of the present review is that it included studies in all languages with no date restrictions, demonstrating the effect of ET at different stages of CVI severity.

## CONCLUSION

Exercise training is a valuable tool for treatment of mild and advanced CVI conditions, since it promotes hemodynamic and musculoskeletal improvements that improve functionality. In individuals with mild CVI, there are also benefits in health-related quality of life parameters, while in the most severe cases there is no evidence to support improvement in venous reflux or health-related quality of life.
